# M. Velpeau

**Published:** 1864-04

**Authors:** 


M. VELPEAU AT THE ACADEMY OF SCIENCES OF PARIS.—This eminent surgeon has just vacated the president's chair of this academy, and it redounds in no small degree to the honor of the professsion that such an eminent post has been held and so worthily occupied by M. Velpeau. It reminds us of Sir B. Brodie presiding at the Royal Society. Our readers are aware that the academy consists of numerous sections, the members of which are eminent in the different branches of science. Among these branches there is of course one for medicine and surgery, and the attainment of a membership in this section is the highest point towards which the imagination of French physicians and surgeons is wont to soar. M. Velpeau delivered, before leaving the chair, a most appropriate and touching speech, in which he especially dwelt on the fact of having started from the humble ranks of life, and having at last obtained the envied honor of presiding, for one year, over the Academy of Sciences of Paris.—*London Lancet, March*, 1864.

